# Supplementary material for: Imaging Minimal Bacteria at the Nanoscale: a Reliable and Versatile Process to Perform Single-Molecule Localization Microscopy in Mycoplasmas
Source: Microbiol Spectr. 2022 May 31;10(3):e00645-22. doi: 10.1128/spectrum.00645-22 (PMC9241803; doi:10.1128/spectrum.00645-22)
Supplement: Supplemental file 1 — Supplemental material. Download spectrum.00645-22-s0001.pdf, PDF file, 1.4 MB [file spectrum.00645-22-s0001.pdf]

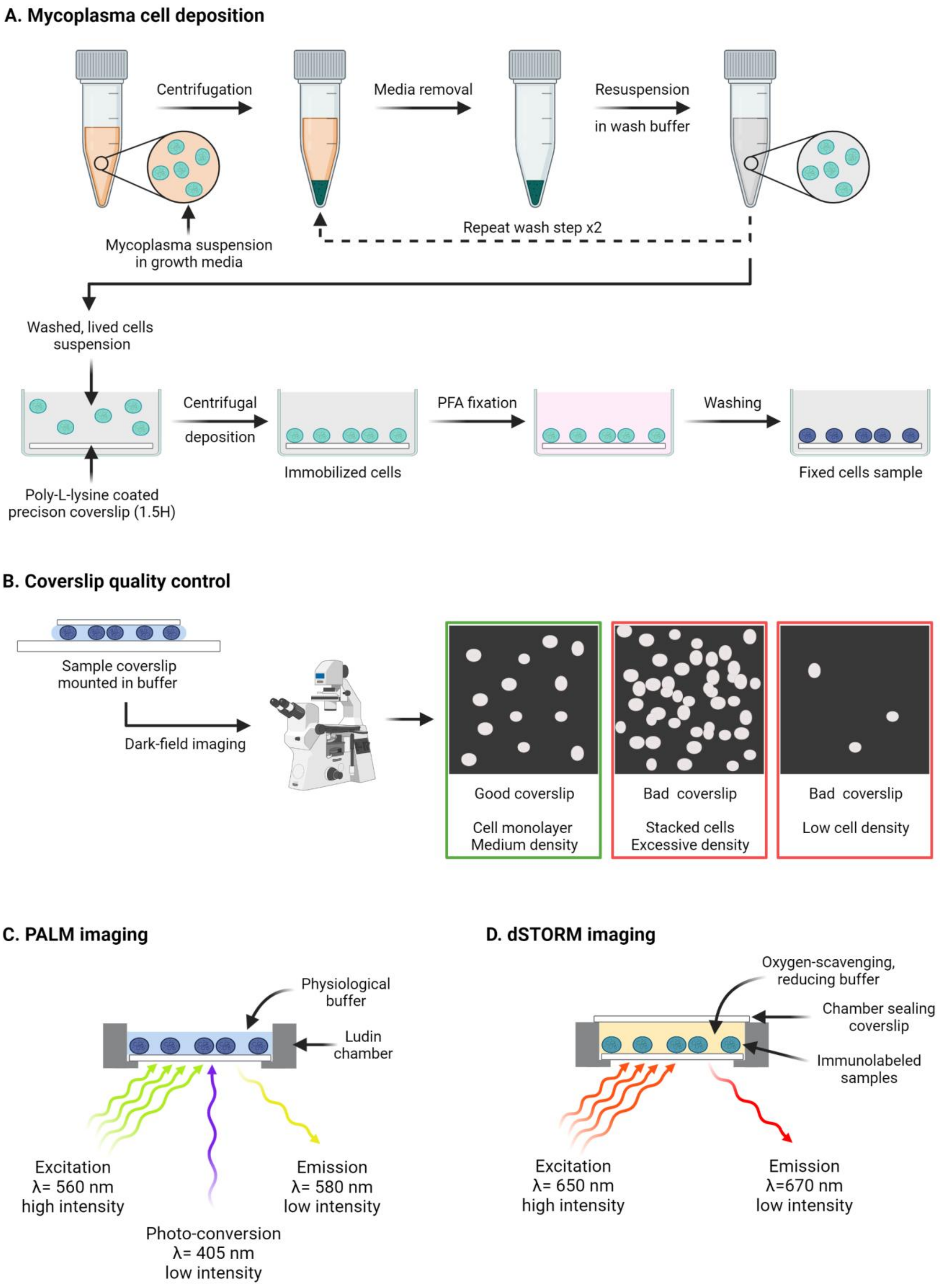

A. Mycoplasma cell deposition
Centrifugation
Media removal
Resuspension
in wash buffer
Mycoplasma suspension
in growth media
Repeat wash step x2
Washed, lived cells
suspension
Centrifugal
deposition
PFA fixation
Washing
Poly-L-lysine coated
precison coverslip (1.5H)
Immobilized cells
Fixed cells sample
B. Coverslip quality control
Sample coverslip
mounted in buffer
Dark-field imaging
Good coverslip
Cell monolayer
Medium density
Bad coverslip
Stacked cells
Excessive density
Bad coverslip
Low cell density
C. PALM imaging
Physiological
buffer
Ludin
chamber
Excitation
λ= 560 nm
high intensity
Photo-conversion
λ= 405 nm
low intensity
Emission
λ= 580 nm
low intensity
D. dSTORM imaging
Oxygen-scavenging,
reducing buffer
Chamber sealing
coverslip
Immunolabeled
samples
Excitation
λ= 650 nm
high intensity
Emission
λ=670 nm
low intensity

Figure S1: Schematic overview of the SMLM samples preparation process. A. Mycoplasma cells deposition and fixation on coverslips. Cells grown in culture media are harvested and washed, then deposited on poly-L-lysine coated coverslips using centrifugation to force the cells to sediment. After PFA fixation, the coverslips can be further processed by immune-labeling or used directly. **B.** Quality control of the sample coverslips. In order to check the proper deposition of cells on the coverslip, and to gauge the cell density, coverslips are mounted on glass slides (cells facing the glass) using a mounting buffer. Dark-field imaging is then performed to assess the samples. **C.** Schematic overview of the PALM imaging process. The coverslip is mounted in a Ludin chamber, filled with PBS buffer. Imaging is performed by providing continuous excitation illumination at medium intensity while simultaneously illuminating the sample with low-intensity ultra-violet light to photo-convert the fluorescent protein. **D.** Schematic overview of the dSTORM imaging process. The coverslip is mounted in dSTORM imaging buffer, in a sealed Ludin chamber. Imaging is performed by providing continuous excitation illumination at high intensity in order to send the fluorophores to their dark state.

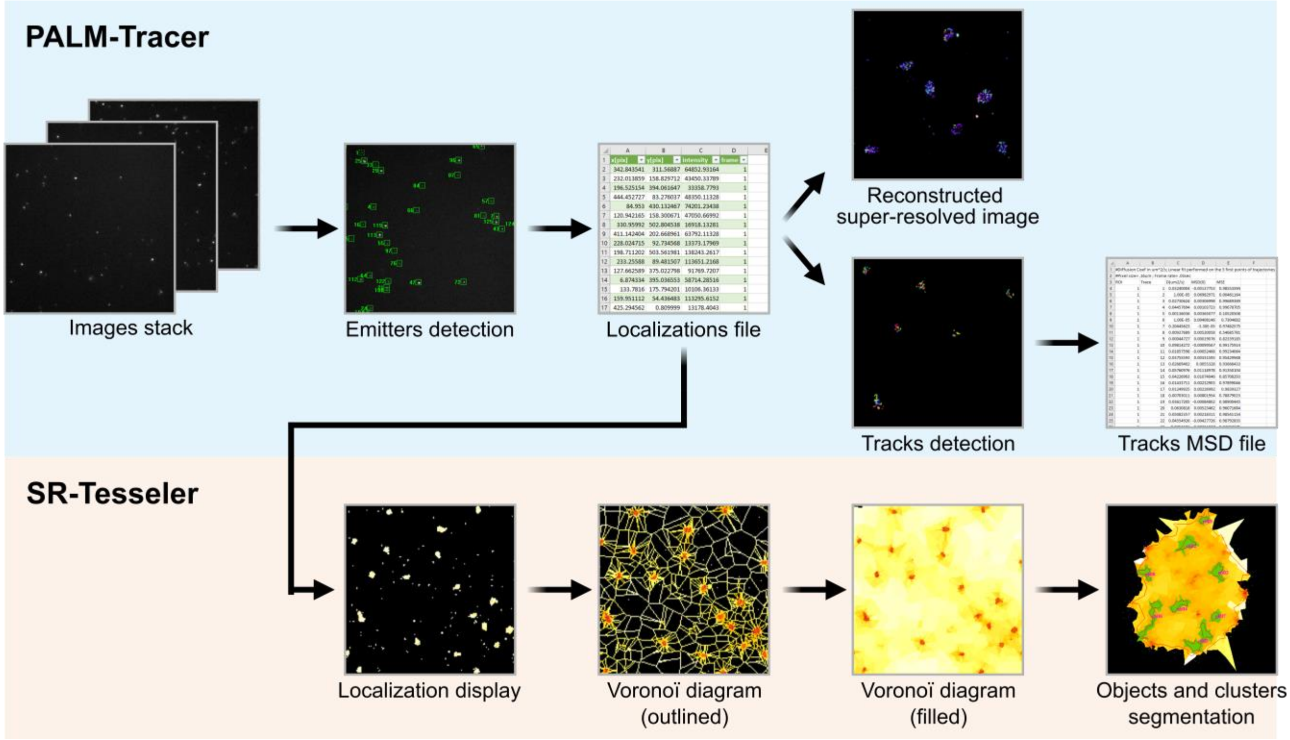

Figure S2. Schematic overview of the SMLM imaging data processing. Images stacks collected during data acquisition are processed using the Metamorph plug-in PALM-Tracer. Emitters are detected on each frame, and their localizations are computed by Gaussian fitting, yielding a localization file indicating for each emitter an x and a y localization value, an intensity value, and a frame number. This localization file is then used to reconstruct a super-resolved image, and to create emitters tracks from which the MSD values are derived. SR-Tesseler is used in parallel to analyze the localization file. First, a Voronoï diagram is computed, based on which objects (red outline) and clusters (green fill) are automatically segmented based on set density, detection and size threshold parameters.

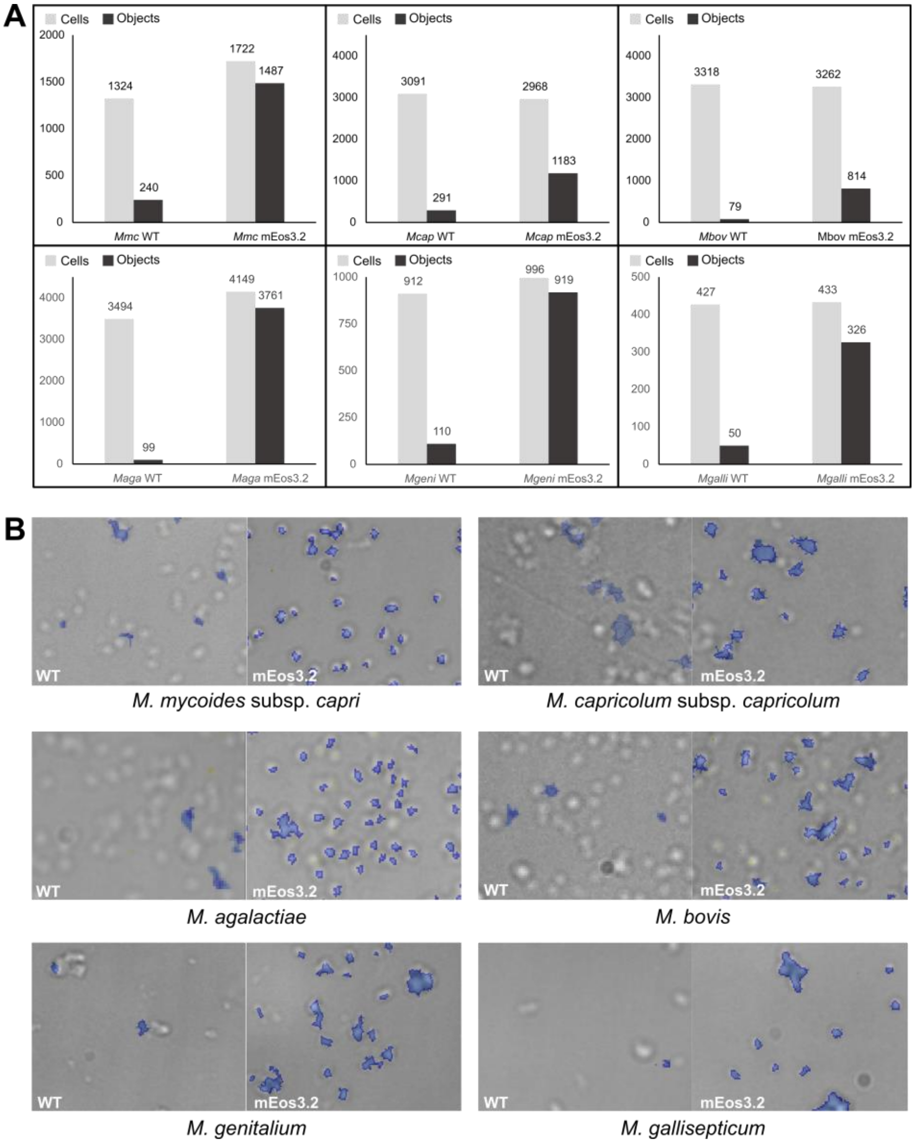

Figure S3: Assessing the functionality of the photo-convertible fluorescent protein mEos3.2 in multiple *Mycoplasma* species. Six mycoplasma species were transformed with the plasmid pMT85-PSynMyco-mEos3.2 and imaged by PALM (see Figure 1). The data presented here correspond to a single representative field of view (512x512 pixels; pixel size: 160 nm). **A.** Comparison of the number of cells and Tesseler segmented objects in the field of view. The bar graph indicates the number of objects segmented by Tesseler (equivalent to a cell) compared to the number of actual cells found in the imaged field. **B.** Localization of the Tesseler objects compared to the mycoplasma cells. Sample images show the overlay of a contrast-phase image and of the objects segmented by Tesseler (blue) in the same field.

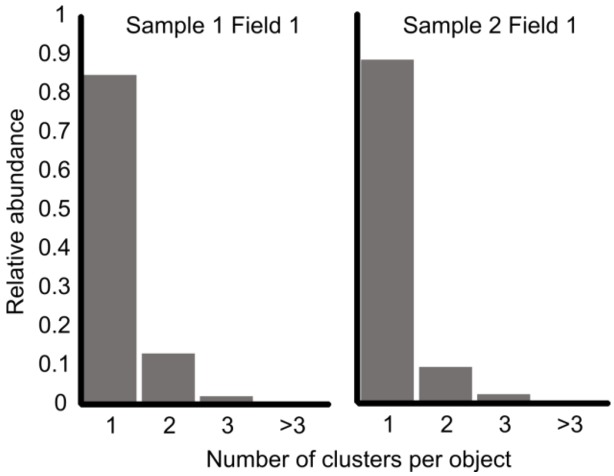

Figure S4: Tesseler clustering of mEos3.2 localizations in the cytoplasm of *Mycoplasma mycoides* subsp. *capri*. *Mmc* pMT85-PSynMyco-mEos3.2 cells were imaged using PALM, and Tesseler clustering of the fluorescence signal was performed. For each dataset, the number of clusters per Tesseler-segmented object was computed. The bar graphs display the distribution of the number of clusters per object. The data presented here correspond to two fields of view (512x512 pixels; pixel size: 160 nm), taken from two independent coverslips.

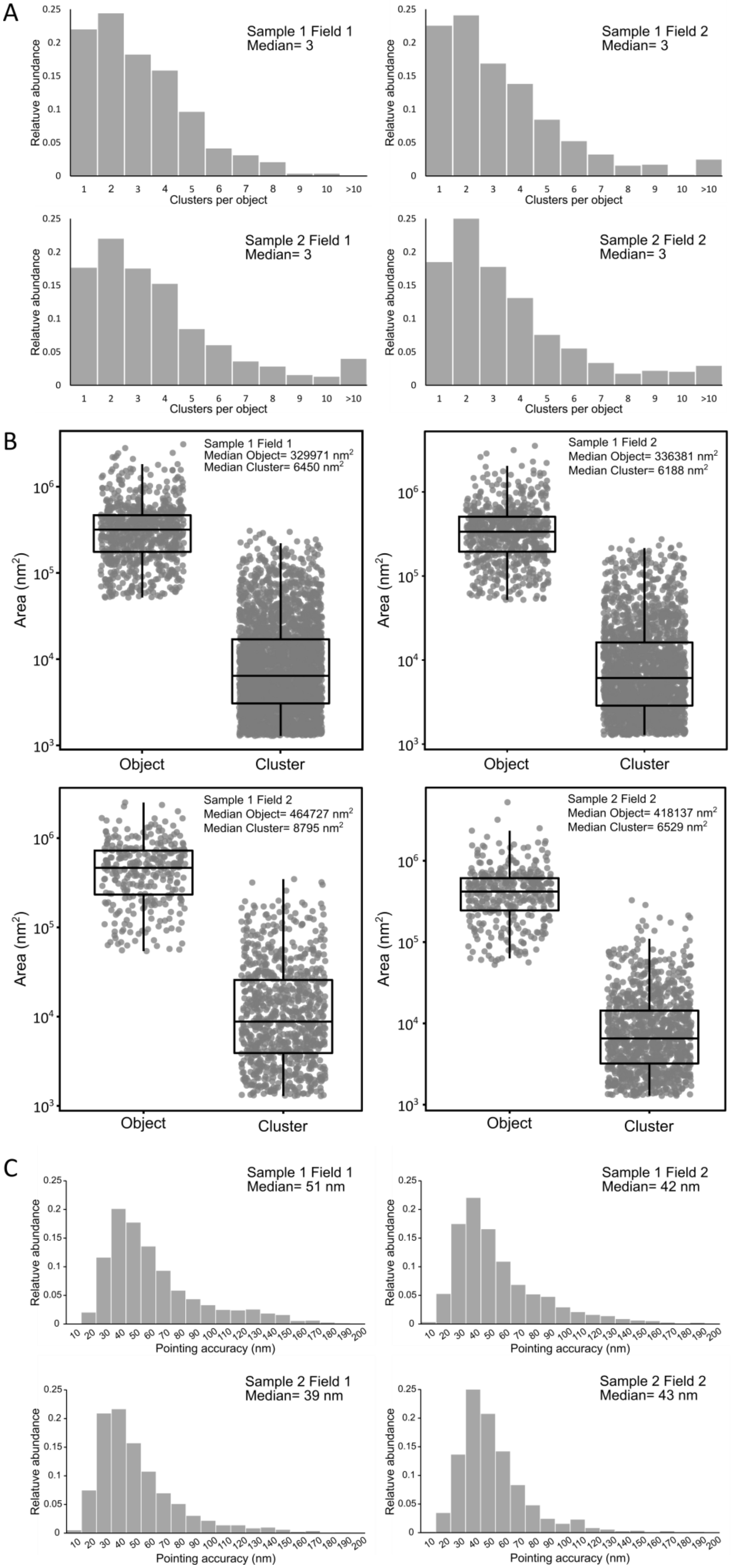
A
Sample 1 Field 1
Median= 3
Sample 1 Field 2
Median= 3
Sample 2 Field 1
Median= 3
Sample 2 Field 2
Median= 3
Relatuve abundance
Clusters per object
B
Sample 1 Field 1
Median Object= 329971 nm²
Median Cluster= 6450 nm²
Sample 1 Field 2
Median Object= 336381 nm²
Median Cluster= 6188 nm²
Sample 1 Field 2
Median Object= 464727 nm²
Median Cluster= 8795 nm²
Sample 2 Field 2
Median Object= 418137 nm²
Median Cluster= 6529 nm²
Area (nm²)
Object
Cluster
C
Sample 1 Field 1
Median= 51 nm
Sample 1 Field 2
Median= 42 nm
Sample 2 Field 1
Median= 39 nm
Sample 2 Field 2
Median= 43 nm
Relatuve abundance
Pointing accuracy (nm)

Figure S5: Replicates of the PALM imaging of an F-type ATPase in *Mycoplasma mycoides* subsp. *capri*. *Mmc* 0575-mEos3.2 cells, expressing a mEos-fused variant of the β-subunit of the ATPase $F_{1\text{-like}}$ domain, were imaged by PALM (see Figure 2). The data presented here correspond to four fields of view (512x512 pixels; pixel size: 160 nm) taken from two independent coverslips. **A.** Tesseler clustering of the fluorescence signal. The number of clusters per Tesseler-segmented objects was computed. The bar graphs display the distribution of the number of clusters per object. **B.** Objects and clusters sizes. The dot plot presents the area (in nm^2) of each object and cluster segmented by Tesseler, to which a boxplot showing the median, interquartile range, minimum and maximum is overlaid. The median value of each data set is indicated. **C.** Evaluation of the PALM imaging pointing accuracy. The bar graphs display the distribution of the pointing accuracy derived from each track. The median value of each data set is indicated.

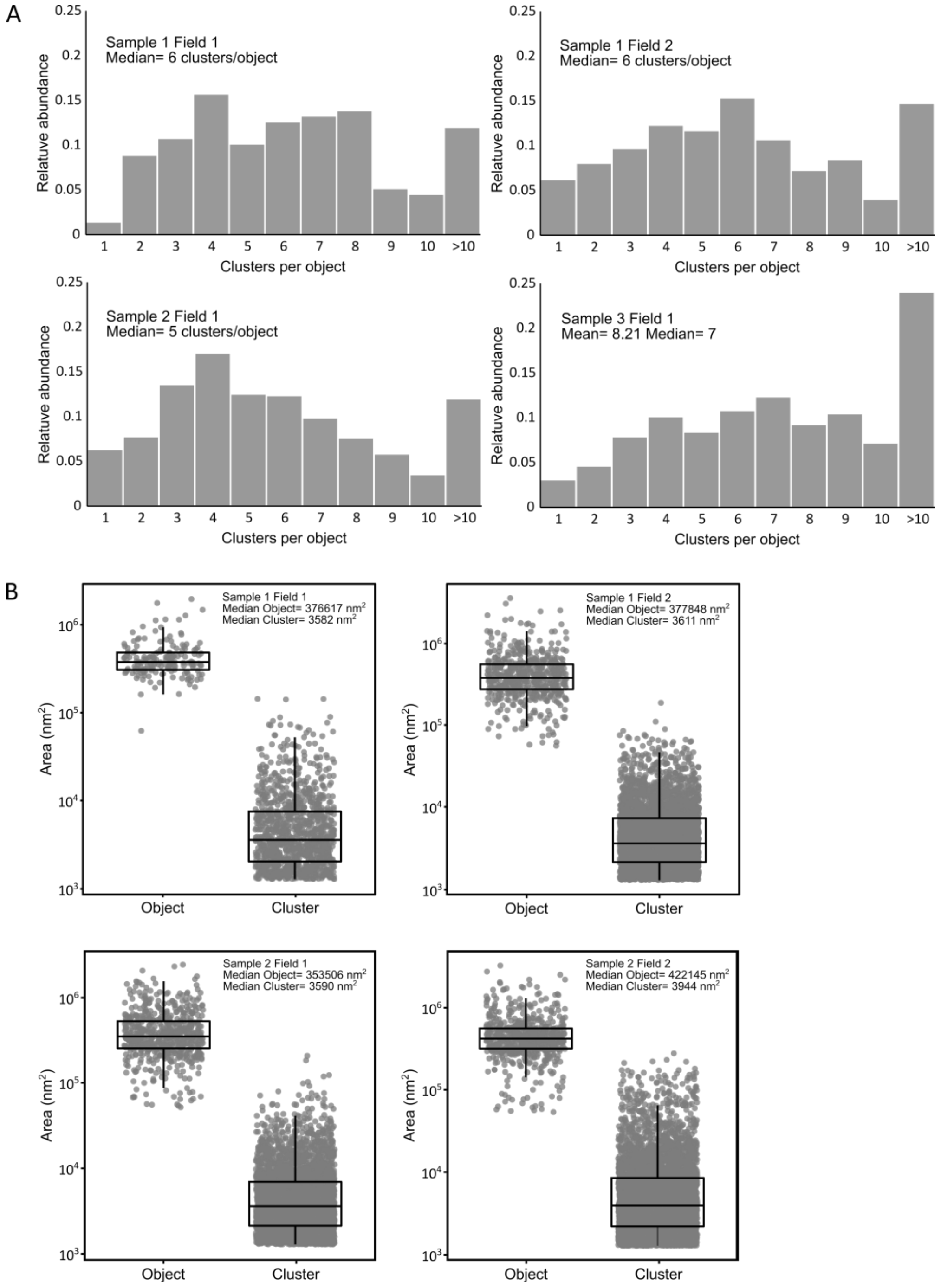

Figure S6: Replicates of the dSTORM imaging of an antibody-specific protease in *Mycoplasma mycoides* subsp. *capri*. *Mmc* 0582-HA cells expressing an HA-tag fused variant of the serine protease MIP_{0582} were immune-labeled and imaged by dSTORM (see Figure 3). The data presented here correspond to four fields of view (512x512 pixels; pixel size: 160 nm) taken from two independent coverslips. **A.** Tesseler clustering of the fluorescence signal. For each field of view, the number of clusters per Tesseler-segmented objects was computed. The bar graphs display the distribution of the number of clusters per object. **B.** Objects and clusters sizes. The dot plot presents the area (in nm^2) of each object and cluster

segmented by Tesseler, to which a boxplot showing the median, interquartile range, minimum and maximum is overlaid. The median value of each data set is indicated.

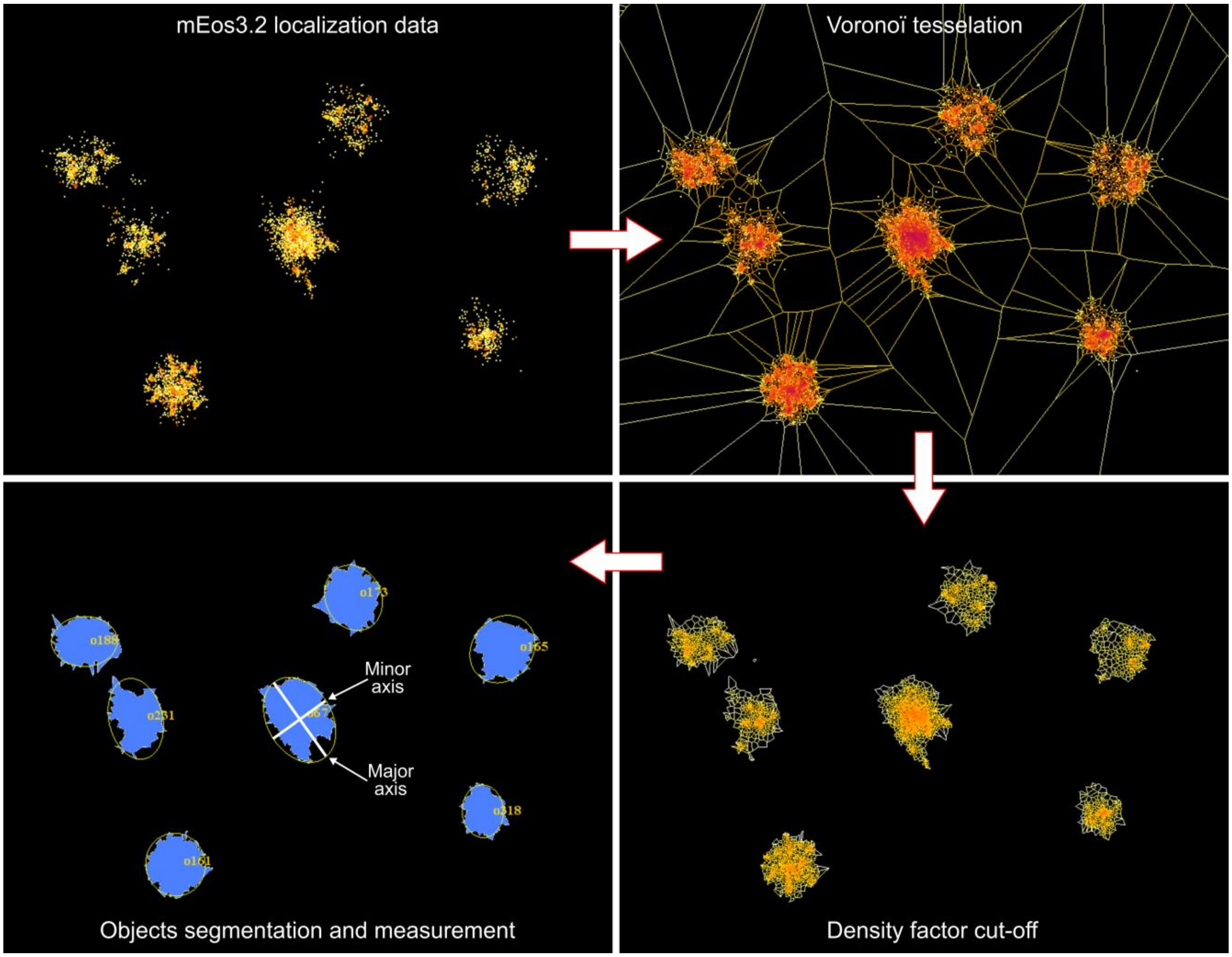

Figure S7: Schematic overview of the cell size estimation from PALM imaging data. Top-left: localization data collected from *Mmc* cells expressing the mEos3.2 as a soluble monomer in the cytoplasm are displayed in Tesseler. Top-right: Voronoï tessellation of the localizations (no density cut-off). Bottom-right: Voronoï tessellation of the localizations is performed again after setting a density factor cut-off (here: 1) in order to filter the signal coming from outside the cells. Bottom-left: Tesseler objects are segmented and their dimensions (major axis and minor axis) are computed.

Supplementary Material:
Fusion proteins coding sequences

>MMCAP2_0582-HA
ATGAAAAGATTGAATAAATTATTGATGTATATATCATCATCAACACTTCTACTCCCGATTACT
TTATTAGTTGCTTGTACTCCTTCTAAAGTAGTTGCTAAACCAATTAATGATGATGAATTTAAC
AAGCTTATTGATTCTATAAAAACTGAAAATGATCTTTTAAAATATGCTGATATTAGATTTAAA
AATCCTAATGGTGCAGATACTAAAAAAGAAGACATAATACCTTCACAACTAAAAAGTGAAA
ATATAAGTATAACTTTCAAAGGTAAATATCAAGGACAAATTAGTGCAGTAGTTACAAATGTA
GATGTTGATAGAACAAACCCTTTTGCAGTACAAAATGAAGCTACAATATTTGTTCAATTTAA
AAATCTTAAAACAAATACATCAAAACCTATAAGTATTACTATTAAAGGATTGAATCAAAAAG
GTAATTTTGATGCTTCTGGGAATATAGTGGTGGATGATTTTGCATATTTTGGTGGACTAGTG
GATATGATGAGTATACTAAGAAAGACCAAAAATCAAGGTTTGATTATGATAATGAAAGATA
TATGACTAGATTGAAAAGTCAATTTGGTAATAGTTCAAATTCAATAAATCTAAAAGAGTATA
GAGGTCTAGAAACAAAGCAAGAAAACATTAAAAAATTTGATGATCAAGCAGCAATTAGTAA
CTTTGACACCTATTATAATGCTGCGTTAAAAGGATTTACTTTACCTGTATATGGTAGTGATGG
AAAAGTAAGCGGTTTAAAAATTTATGAAGGAGCAGAAATAGGAAAAGGTCCTTCAGTTGTTG
ACTCACTAGGTAGAAATGAAAAAGCTAAAACTGTTGGATTAGCTAGAACTTTACCTAATGAA
GAGTATAAAACATCAGCTATTCAGACTTTTCAAACAAACTTTACAATCTATAAAGATTATGA
AAAAGAGATAGAAGAAGCAGAAGATAACATAAAATTATTTGATTCTTGAAATGAACAACAA
ATTCAAAGTTATATTTCAGCACAGTTAACACAATTAAGGCTAAATTATGAGGATGAAGTTTCT
CAAATAGATAGAGAAATATCTCAAACACAACCTGATAAAACAACTATACTATCAAATCTTAA
TCAAAAGAAATCAAAGATAGAATCTGAGTATCAAAAAGAGTTGTCAACAATATCAAAATTA
AACAAAGATAGTCTTAAAGAATGGCAAAGAAAAGAAATTGAAAAATATAATGAAAAGAAA
AAAGAAAAAACATTTCAAATTTCTGAATCTGGAACAATGTGAATAATGGATTATTTAGATGA
AAATGCTGGGAAGAATCCAACTAAATTTTACTTTGGAACTAACTCACACGTTGCTAAAGGAA
TTAAAGATGGTATGGTTTCTTTCTCTTTAACTAGATTAAATTCAGAGGTTAAAGTAGGTCAAA
CATTTAAATTAAATGGGCATGATAGCAATTTTACTAAATTTACATTTTCTCCAATTAATGGTA
ATAAACTTGAAGATGCTGTTACTGCAATTTTTCATGCAACTGATTTTATAAATGAAAATAGCA
GTCCACTTAAATTACTAGATTCTGAGCAAAAGTCTAAATATAATGGCGCTGGGATATTTGCTG
ATTTTGCTATTGTTGAAGTTGATTTTGCAAAACTACTAGACAAAGGCAAATATTCATATTCTG
TGTGAAGCGCTTCTAATGATATAACAAATCAATATGAAACTGAGCAAAATAAATTAATTAGC
AAAATAACTAATAATTACTCAGAAAGTGATAAAAAGGTTAAGTTTTTCTCTGATTCTTTATTA
AACGAACAAACTTATGCTAAATTTGATAGACCACTTGATTTTGATCCTAAAAAAGAAGATGA
ATTGAAAAAATATAACGATTTAGATAGTCTATACATAGTTGGTTATCCTACAGCTTATAAAG
ATTTTTATTTAGATCAGTATGAAGATGAAAAACAACTAAAAAATAAAAAATATGATTTTTCA
TTGTGAATTAATAGTGAGTATAAATTCTATAATAAACTAATTAATAAGGAAGGTTCAACTAA
TTCATTTAAAGAATATGAAACAGGAAAAGGTAATTTCTTTTCATACCAAATTGGATATCGTTC
ATTTATTGATAAGCCAGGATTGACTGATGCTTTTATAACTGTTAACAAAGTCGGTAAAAAATT
ATATTCATTAAAAGATAAAAATAAAAATGAAGTTAAAAAATACTTCAATTATGGTTTAGAAA
TACTTCCAAGATTTTATGCACCAGCTGGTGGAGCTTCTGGTTCTAGTGTTAGAACTAAAGATA
ATAAATTACTAGCTGTATATCACGCATCAAACGAAACAGCTAGAACTGGTTTAGCTGTTGCA
TTTAGATCTGATGGTTATGATTATAAAAACTTATTTGGTGATTATAAATTAGGTCAATATGAT
TTAATTTATGGTGGTGGAAAAGACCAACAAAAAGAAAAATCATATAGAGAAGTTATGAATA
AAATGTATAGCGGTAAGAAAAGCGCATTATTCCAAAATGGATTTACTGATGACAAAATCCCA
TCTGAATTTAAATTCAATAATTCAACACAAAACTATCCATATGATGTTCCTGATTATGCTTAG

>MMCAP2_0575-mEos-Nt
ATGTCAGCTATTAAACCAGATATGAAAATTAAATTAAGAATGGAAGGAAATGTAAATGGAC
ATCATTTTGTAATAGATGGAGATGGTACTGGTAAACCATTTGAAGGTAAACAATCAATGGAT
TTAGAAGTTAAAGAAGGTGGTCCATTACCATTTGCTTTTGATATTTTAACTACTGCTTTTCATT
ATGGTAATAGAGTTTTTGCTAAATATCCAGATAATATTCAAGATTATTTTAAACAATCATTTC
CAAAAGGTTATTCATGAGAAAGATCATTAACTTTTGAAGATGGTGGTATTTGTAATGCTAGA
AATGATATTACTATGGAAGGTGATACTTTTTATAATAAAGTTAGATTTTATGGTACTAATTTT
CCAGCTAATGGTCCAGTTATGCAAAAAAAAACTTTAAAATGAGAACCATCAACTGAAAAAAT
GTATGTTAGAGATGGTGTTTTAACTGGTGATATTGAAATGGCTTTATTATTAGAAGGTAATGC
TCATTATAGATGTGATTTTAGAACTACTTATAAAGCTAAAGAAAAAGGTGTTAAATTACCAG
GTGCTCATTTTGTTGATCATTGTATTGAAATTTTATCACATGATAAAGATTATAATAAAGTTA
AATTATATGAACATGCTGTTGCTCATTCAGGTTTACCAGATAATGCTAGAAGAGGATCAGGA
TCAGGATCAAACGGAAAAATTTTAAGTATATCTGGTGATGTAATTGAAGTACAATTTGAAAA
AAGCAATTTGCCTTCAATTAATCACTTATTAACAACTCATGATAATCAAACATATTTATTAGT
AAAAAGTGTTATTAATGAAACTAACATTAAAGCAATCATCATTTATGCATATAAACAAATTT
CACTAAGCGATGAAATTATAAATACTAATAAAAGTTTTATGGTTCCAGTAGGAAGTAAAGCA
AAAAACAACATTTTTAGTTTTACTGGAATTTCACTAAACAATAAAAAAGATGATAAACAAGA
ATATGTTGAAATGAATTCAACTATTAATAATAAAAGAGAATTAAGCACTGAATTTGAACTAA
TTGAAACAGGTATTAAAGCGATTGACTTTTTTATTCCAATTTTTAAAGGATTTAAATTAGGAA
TTTTTGGTGGAGCTGGTGTTGGTAAAACAGTTTTAATGAAAGAAATCATTTTTAATGTTAATA
ATAAATATAAAAACACTTCAAATATTTTTATTGGATCAGGTGAACGTTCAAGAGAAGGTATT
GAACTTTATGATGAACTAACTGAATCTAATTTAATGAAAAATTCAACTATGTTTGTTTCTAAA
ATGAACGAATCTCCAGGAGCACGTATGTCAATTGTTCCAATTGGAGTTACTGCTGCTGAGTAT
TTAAGAGATGTTAAAAAAGAAGATGTTTTATTATTTATTGATAATATTTATCGTTTTATTCAA
GCAGAAAATGAAGTAAGTGCTACACTTGGTAAAAAACCTTCAGTTGGTGGGTATCAATCTAC
ACTAGAAAGTGATGTTGCAAATATTCAAGATCGTTTATTTAAAAATAAAAACGGAGCGATTA
CTTCATTTCAAACTGTGTTTTTACCAATGGATGATTTAAGTGATCCATCAGCTGTAGCTGTATT
TAATCATTTAGATTCAAATCTAGTTTTATCAAGAGATCAAGCTGCAAAAAATATTCTTCCTGC
TTTTGATCCACTAGCAAGTTCTTCTAGTTCAGTTGATGAAACTTTAATTGGTAAAAAACACTT
TAATGCCATTATTGAAGTTAAAAGAATTTTAAAAGCTTATAAAGATTTAGAAGATGTTATTTT
AATTTTAGGATTTGATGAATTAGATGCTGAAAGTAAAATTTTAGTTAAAAAAGCTTTACAATT
AGAAAATTTCTTTACTCAATATTTCTTTATGACTGAACAATTTACAAAACAAAAAGGTCAATA
TGTACCTTTAAATGAAACAATTGATAGTGTTATTAGAATTATTGAAGGAAAATATATTAAAC
AATCTCCTGAAATCTTTTCATATATTGGTTCAGCTTTAGATTTAAAAACTGATGAAGAATTAG
GATTATAA
